# Evaluating the Impact of Year-Long, Augmented Diabetes Self-Management Support

**DOI:** 10.1089/pop.2018.0175

**Published:** 2019-12-02

**Authors:** Benjamin M. Bluml, Leslie E. Kolb, Ruth Lipman

**Affiliations:** ^1^ American Pharmacists Association Foundation, Washington, District of Columbia.; ^2^ American Association of Diabetes Educators, Chicago, Illinois.; ^3^ American Dental Association, Chicago, Illinois.

**Keywords:** diabetes support, diabetes education, Federally Qualified Health Center, telephonic support, patient engagement, self-management

## Abstract

This was a randomized controlled study to test a scalable intervention model addressing the need for ongoing diabetes support. The study included individuals receiving care in a Federally Qualified Health Center (FQHC) with HbA1c >8. The aim of this project was to determine whether augmenting diabetes self-management education (DSME) with support for an economically vulnerable population might better meet patient needs and reduce morbidity and premature mortality. The intervention utilized pre and post comparisons and was designed to test the efficacy of a telephonic diabetes support intervention to increase patient engagement in self-care and with the health care system as a means to improve clinical outcomes. There were significant improvements in HbA1c, body mass index, low-density lipoprotein cholesterol, triglycerides, and depression screening scores in the year following DSME. However, there was no statistically significant difference between the 2 groups. This randomized controlled study demonstrated that comprehensive face-to-face care with consistent assessment and documentation over time in FQHCs produce clinically significant and predictable improvement for people with diabetes. The addition of structured provision of telephonic support overlapping in time with the comprehensive face-to-face process of care in this environment did not produce statistically significant clinical or behavioral care improvement.

## Introduction

Diabetes is a medically complex condition that requires multifaceted approaches to treatment, of which self-management is an essential element. The American Association of Diabetes Educators (AADE) supports the AADE7 Self-Care Behaviors, which consist of healthy eating, being active, taking medications, monitoring, reducing risks, problem solving, and healthy coping.^1^ Controlling blood pressure, cholesterol level, and glycemia require not only appropriate use of prescribed medications but ongoing monitoring of food consumption and engagement in physical activity.

Patient support has received particular attention as an element with potential to be particularly useful in diabetes care.^2^ Diabetes Self-Management Education and Support (DSMES) is the ongoing process of facilitating the knowledge, skills, and ability necessary for diabetes self-management, as well as activities that assist a person in implementing and sustaining the behaviors needed to manage his or her condition on an ongoing basis, beyond or outside of formal self-management training.^3^ The support component in DSMES can engage individuals with limited specific health care training, such as community health workers (CHWs), to interact with the person with diabetes to aid with diabetes decision making in concert with health care professionals. CHWs serve as a conduit to bring insights back to the health care team and help to identify challenges associated with the individual's self-management plan.

A variety of models have been proposed to provide the support required to achieve the desired improvement in diabetes knowledge and self-management skills. CHWs, peer support, and online programs can help address issues having to do with socioeconomic conditions, as well as aid in health care system navigation.^4–6^

AADE sought to test a scalable intervention model to address the need for ongoing diabetes support for individuals receiving care in a Federally Qualified Health Center (FQHC) with HbA1c >8, meaning that they were above the Healthcare Effectiveness Data and Information Set definition of glycemic control.^7^ The study was conducted to answer the question of whether augmented diabetes self-management support with an economically vulnerable population addresses unmet needs and reduces morbidity and premature mortality.^8^

## Methods

### Program description

A randomized clinical trial registered at ClincialTrials.gov # NCT02160639 was conducted at 4 sites. A total of 446 individuals were enrolled (Table 1). The intervention utilized pre and post comparisons. The study was designed to test the efficacy of a telephonic diabetes support intervention to increase patient engagement in self-care utilizing the health care system as a means to improve clinical outcomes. The study was reviewed and approved by Schulman Institutional Review Board (Blue Ash, OH). Study participants provided written informed consent.

**Table 1.** Demographics and Baseline Comparison of Control and Intervention Groups

	*Total*		*Control*		*Intervention*			
	*N*	*%*	*N*	*%*	*N*	*%*	*Chi-square*	t *Test*
Total	446	100	225	50.4	221	49.6	*P*	*P*
Completed Study								
No	258	57.8	136	60.4	122	55.2	0.262	
Yes	188	42.2	89	39.6	99	44.8		
Sex								
Female	261	58.7	135	60.00	126	57.3	0.559	
Male	184	41.3	90	40.00	94	42.7		
Ethnicity								
White	218	48.9	110	48.9	108	48.9	0.650	
African American/Black	98	22.00	49	21.8	49	22.2		
Hispanic	115	25.8	58	25.8	57	25.8		
Native American	12	2.7	5	2.2	7	3.2		
Other	2	0.4	2	0.9	0	0.00		
Not Specified	1	0.2	1	0.4	0	0.00		
Preferred language								
English	286	82.7	141	82	145	83.3	0.739	
Spanish	60	17.3	31	18	29	16.7		
Smoking Status								
Nonsmoker	44	31.2	23	33.8	21	28.8	0.120	
Never smoked	43	30.5	15	22.1	28	38.4		
Current smoker	49	34.8	26	38.2	23	31.5		
Within last 12 mo.	5	3.5	4	5.9	1	1.4		
Depression Status (PHQ2)								
Depressed	81	31	40	33.1	41	29.3	0.511	
Not Depressed	180	69	81	66.9	99	70.7		
Age in years mean (SD)	336	54.4 (10.6)	167	54.2 (11.2)	169	54.6 (10.0)		0.778
Height in inches mean (SD)	277	66.0 (4.1)	141	65.7 (4.4)	136	66.2 (3.8)		0.269
Weight in lbs. mean (SD)	295	228.3 (63.4)	147	224.2 (67.2)	148	232.3 (59.3)		0.270
BMI mean (SD)	324	36.8 (9.3)	158	36.4 (9.2)	166	37.1 (9.5)		0.508
Hemoglobin A1c mean (SD)	340	10.2 (1.7)	168	10.1 (1.7)	172	10.4 (1.7)		0.195
LDL cholesterol mean (SD)	237	99.7 (37.6)	117	99.9 (38.3)	120	99.6 (37.1)		0.937
HDL cholesterol mean (SD)	240	42.6 (12.6)	119	44.2 (14.7)	121	41 (9.9)		0.051
Triglycerides mean (SD)	240	235.3 (214.4)	119	237.2 (224.8)	121	233.5 (204.6)		0.896
Total cholesterol mean (SD)	240	185.9 (50.2)	119	188.4 (52.3)	121	183.4 (48.1)		0.445
PHQ2-scores mean (SD)	259	1.6 (1.8)	120	1.9 (1.9)	139	1.4 (1.9)		0.021
Systolic BP mean (SD)	322	134 (19.6)	155	136.5 (20.4)	167	132.9 (18.7)		0.096
Diastolic BP mean (SD)	322	78.2 (11.3)	155	78.8 (10.2)	167	77.6 (12.2)		0.361

Study participant demograhics and baseline comparison using chi-square analysis of control and intervention groups. Clinical data at baseline were compared using independent sample *t* test for continuous outcomes.

BMI, body mass index; BP, blood pressure; HDL, high-density lipoprotein; LDL, low-density lipoprotein; PQH-2, Patient Health Questionnaire; SD, standard deviation.

### Study population

To be eligible, individuals needed to be either English- or Spanish-speaking adults, ages 21–85 years, and have a diagnosis of type 2 diabetes mellitus. It was required that study participants had not participated in a diabetes self-management education (DSME) program in the last year, and had a HbA1c >8 at the time of enrollment. Subjects excluded from the study included individuals who were currently pregnant or undergoing cancer treatment; had a diagnosis of end-stage renal disease or serious mental illness; or were receiving systemic treatment with prednisone or immunosuppressant therapy following organ transplant.

Randomization was centralized at the AADE office in Chicago to ensure allocation concealment. When it was determined that individuals were eligible to participate in the study and had consented, a call was placed to the AADE office where the blocked randomization chart for the center was used to assign the participant the next consecutive treatment condition. The individual was placed either in a standard DSME program or a standard DSME program augmented with the telephonic support provided by CHWs, known as health navigators. Demographics of study participants are shown in Table 1. All study participants were receiving care at one of the 4 FQHC study sites.

Telephonic support was provided through the National Center for Farmworker Health, Inc. (NCFH), which served as a centralized call center for all 4 sites. Training was provided for health navigators, covering DSME delivery and data entry. On-site training of health navigators at NCHF was conducted by 2 credentialed diabetes educators. Prior to the live training session, the health navigators at NCFH took the AADE's online Fundamentals of Diabetes course and Motivating Behavior Change recorded webinar.

### Data management

A data set was standardized across all participating sites and a common data collection system, the Project IMPACT Database Explorer, was utilized by health care providers and health navigators to streamline data organization and reporting. The system was developed by the American Pharmacists Association Foundation as an extension of their work in Project IMPACT: Diabetes.^9,10^ It provided secure, role-based access for participant documentation of measures outlined in the following section. The intervention group received regularly scheduled outbound phone calls from an NCFH health navigator who had access to information about the individual and her or his participation in DSME through the Project IMPACT Database Explorer system. Initiation of outbound calls occurred and was documented while participants were engaged in the DSME process to ensure continuity between education and support. Calls were scheduled at a convenient day and time for the individual participant every 2 weeks for 3 months. The frequency of calls was then decreased to 1 call per month. The initial focus of the conversations during each call was a discussion about the lessons learned and goals set during DSME.

### Measures

Demographic information collected on study participants included age, sex, and race. In both groups, participation in diabetes education sessions was tracked and in the intervention group, the number of telephonic support contacts was documented. Clinical measures monitored were fasting blood glucose, HbA1c; systolic and diastolic blood pressure; low-density lipoprotein (LDL) cholesterol, high-density lipoprotein (HDL) cholesterol, and triglycerides from which total cholesterol was calculated; height and weight from which body mass index (BMI) was calculated. In addition, participant well-being was monitored over time using the Patient Health Questionnaire. Information on process measures was collected to compare engagement in the control and intervention groups. This included participation in dental exams, eye exams, and foot exams; whether participants had had an influenza or pneumococcal vaccine; and the total number of visits to the participants' FQHC. The number and category of AADE7 Self-care Behaviors goals set during the study period as well as the number and category of self-care goals achieved also were recorded. Participant satisfaction was evaluated pre and post intervention using a 6-question survey, which was graded using a Likert score from 1 to 5.

This study was designed to examine efficacy of an intervention in a real-world situation. Therefore, the data used for the 1-year follow-up visit were taken from the visit that was closest to 365 days after baseline but was accepted from visits between 6 and 18 months following the baseline visit.

### Statistical analysis

Descriptive statistics for the control and intervention groups were compared using chi-square analysis and are shown in Table 1. Baseline clinical data for the control and intervention groups were compared using independent sample *t* test for continuous outcomes and none were found to differ (Table 1).

Changes in clinical outcomes over time between the 2 groups were compared using repeated measures analysis of variance (ANOVA). Table 2 presents the measurements taken at baseline and 1 year along with *P* values for main effects and interactions for each of the repeated measure ANOVAs. Mann-Whitney U tests were used to compare the tallies for the total number of each process measures (Table 3). Chi-square tests were used to compare the number of self-care goals set and achieved (Table 4). Pearson correlation coefficients were used to measure the number of telephonic support contacts with success in achieving the set goal for each of the AADE7 self-care behaviors (Table 5) as well as for responses to the questions in the patient satisfaction survey, which were scored using a Likert score from 1 to 5 (Table 6).

**Table 2.** Baseline and 1-Year Outcomes for Control and Intervention Groups

	*Control*			*Intervention*			P *values*		
	*N*	*Mean*	*SD*	*N*	*Mean*	*SD*	*Group*	*Time*	*Interaction*
Baseline A1c	168	10.1	1.7	172	10.4	1.7	0.567	<0.001	0.207
1-Year A1c		8.7	1.8		8.7	1.9			
Baseline BMI	158	36.4	9.2	166	37.1	9.5	0.455	0.011	0.517
1-Year BMI		36.0	9.1		36.9	9.1			
Baseline HDL	118	44.3	14.8	119	41.1	10.0	0.212	0.393	0.077
1-Year HDL		43.7	13.6		42.9	14.8			
Baseline LDL	111	100.5	38.5	114	98.9	36.1	0.690	0.015	0.980
1-Year LDL		94.9	38.2		93.2	32.7			
Baseline Triglycerides	118	237.4	225.8	120	231.5	204.3	0.658	0.004	0.741
1-Year Triglycerides		208.2	172.7		194.9	141.6			
Baseline Total Cholesterol	118	188.8	52.3	120	183.3	48.3	0.445	0.079	0.001
1-Year Total Cholesterol		175.0	46.4		172.3	40.5			
Baseline Systolic BP	155	136.5	20.4	167	132.9	18.7	0.203	0.217	0.211
1-Year Systolic BP		133.5	16.8		132.9	18.0			
Baseline Diastolic BP	155	78.8	10.2	167	77.6	12.2	0.252	0.842	0.987
1-Year Diastolic BP		78.9	10.7		77.8	10.7			
Baseline PHQ-2	120	1.9	1.9	139	1.4	1.9	0.377	0.003	0.031
1-Year PHQ-2		1.3	1.7		1.4	1.9			

Baseline measurements and 1-year outcomes for control and intervention groups with *P* value main effects and interactions for each of the repeated measures utilizing a 1-way analysis of variance.

BMI, body mass index; BP, blood pressure; HDL, high-density lipoprotein; LDL, low-density lipoprotein; PQH-2, Patient Health Questionnaire; SD, standard deviation.

**Table 3.** Comparison of Control and Intervention Groups Process Measures Over Time

	*Control (N = 225)*		*Intervention (N = 221)*		
	*Mean*	*SD*	*Mean*	*SD*	P
Dental Exams	0.1	0.3	0.2	0.5	0.239
Eye Exams	0.9	0.9	0.9	1.0	0.472
Foot Exams	1.4	0.9	1.4	0.9	0.259
Influenza Vaccination	0.9	0.9	1.00	0.8	0.205
Pneumococcal Vaccination	0.5	0.6	0.5	0.6	0.740
Number of Visits	3.6	1.7	3.9	1.7	0.036

SD, standard deviation.

**Table 4.** Self-Care Goals Set and Achieved

	*Total*		*Control*		*Intervention*		
*AADE self-care goals set*	*N*	*%*	*N*	*%*	*N*	*%*	P
Healthy Eating	327	73.8	159	71.6	168	76.0	0.293
Being Active	237	53.5	111	50.0	126	57.0	0.139
Monitoring	227	51.2	116	52.3	111	50.2	0.670
Taking Medications	197	44.5	106	47.7	91	41.2	0.164
Problem Solving	101	22.8	43	19.4	58	26.2	0.085
Reducing Risk	169	38.1	80	36.0	89	40.3	0.359
Healthy Coping	62	14.0	28	12.6	34	15.4	0.400

	*Total*		*Control*		*Intervention*		
*AADE self-care goals achieved*	*N*	*%*	*N*	*%*	*N*	*%*	P
Healthy Eating	142	43.4	64	40.3	78	46.4	0.260
Being Active	86	36.3	31	27.9	55	43.7	0.012
Monitoring	94	41.4	44	37.9	50	45.0	0.277
Taking Medications	90	45.7	42	39.6	48	52.7	0.065
Problem Solving	63	62.4	29	67.4	34	58.4	0.366
Reducing Risk	90	53.3	39	48.8	51	57.3	0.266
Healthy Coping	28	45.2	10	35.7	18	52.9	0.175

Comparison of control and intervention gourps utilizing chi-square tests of the above AADE-7 self-care behaviors set and achieved.

AADE, American Association of Diabetes Educators.

**Table 5.** Correlation Between Number of Telephonic Interventions and Achieving American Association of Diabetes Educators Self-Care Goal Set

*Self-care goal*	*N*	r	P
Healthy Eating	169	0.319	<0.001
Being Active	127	0.351	<0.001
Monitoring	111	0.335	<0.001
Taking Medications	92	0.344	0.001
Problem Solving	58	0.11	0.411
Reducing Risk	89	0.21	0.048
Healthy Coping	34	0.379	0.027
Total Goals set	680	0.374	<0.001
Total Goals Achieved	222	0.428	<0.001
% goals achieved	32.65%	0.458	<0.001

Correlations between self-care measures and number of telehealth interactions (inbound and outbound contacts). Pearson correlation coefficients, column *r* in the table above, are used to measure association between the number of telehealth interactions, the N column, and the subject achieving the self-care goal. Each subject in the intervention received telehealth interactions on variable self-care goals (ie, not all subjects received all 7 interventions).

Correlation also was measured between the entire intervention group (N = 222) and 3 variables: total goals set, total goals achieved, and % of goals achieved.

**Table 6.** Satisfaction Score Comparisons^*^

	*Control*			*Intervention*			P *values*		
	*N*	*Mean*	*SD*	*N*	*Mean*	*SD*	*Group*	*Time*	*Interaction*
Baseline Total Satisfaction	107	26.70	3.05	136	26.73	3.11	0.548	<0.001	0.504
1-Year Total Satisfaction		28.22	2.52		28.54	2.45			
Baseline Q1	108	4.46	0.59	136	4.44	0.63	0.956	<0.001	0.580
1-Year Q1		4.76	0.43		4.79	0.45			
Baseline Q2	108	4.36	0.57	136	4.43	0.55	0.142	<0.001	0.772
1-Year Q2		4.62	0.59		4.71	0.52			
Baseline Q3	108	4.38	0.68	136	4.49	0.56	0.187	<0.001	0.414
1-Year Q3		4.73	0.45		4.76	0.43			
Baseline Q4	108	4.39	0.53	136	4.43	0.54	0.165	<0.001	0.418
1-Year Q4		4.63	0.54		4.74	0.46			
Baseline Q5	108	4.32	0.67	136	4.24	0.87	0.946	<0.001	0.162
1-Year Q5		4.69	0.59		4.76	0.51			
Baseline Q6	107	4.51	0.57	136	4.52	0.61	0.858	<0.001	0.687
1-Year Q6		4.81	0.39		4.79	0.5			

^*^ Items originally scored on a Likert scale from 1 (*strongly agree*) to 5 (*strongly disagree*). In order to make higher scores mean “greater agreement,” these items were reverse scored. Current scores are on a Likert scale from 1 (*strongly disagree*) to 5 (*strongly agree*).

Q, quarter; SD, standard deviation.

## Results

The mean age of the participants was 54.6 years in the intervention group and 54.2 years in the control group. Distribution of sexes was similar between groups (Table 1). The groups did not differ significantly by race, with almost half of the total study population being white, followed by Hispanic, African American/black, and Native American, with Other and Not Specified accounting for less than 1%.

There were significant improvements in HbA1c, BMI, LDL, triglycerides, and depression screening scores in the year following DSME. However, there were no statistically significant difference-in-differences between the groups for HbA1c, BMI, HDL, LDL, triglycerides, and blood pressure (Table 2). No significant change in either systolic blood pressure or diastolic blood pressure were observed following DSME for either group. Total cholesterol and depression screening score seemed to show a significant group by time interaction; *P* = 0.001 and *P* = 0.031, respectively.

Comparison of engagement in individual process measures associated with the AADE7 self-care behavior of reducing risks did not differ for participants in the intervention and control cohorts (Table 3). Total number of FQHC visits for individuals in the intervention group was statistically greater than for those in the control cohort, *P* = 0.036.

When examining goal-setting preference, there were no differences in the proportion of goals set in any of the AADE7 categories between the groups. However, a significantly greater number of those in the intervention group successfully met their goal for being active: *P* = 0.012 (Table 4). Interestingly, for 6 of the 7 AADE7 self-care behaviors as well as for the total goals set, there was a positive correlation between number of telephonic support contacts and success with attaining the goal (Table 5).

Patient satisfaction as reflected in individual or total responses to the questions in the survey did not differ between the 2 groups. However, both the individual and total responses from all the study participants indicated a statistically significant improvement in patient satisfaction following their participation in DSME, *P* < 0.001 (Table 6).

## Discussion

Individuals in both the usual care control and telephonic support intervention group showed statistically significant and clinically meaningful improvement in HbA1c after participation in this study. This is important because although the study team set out to recruit those with an HbA1c >8%, in fact the majority of the individuals participating in this study were categorized with poorly controlled glycemic status at enrollment (eg, HbA1c >9%). These findings reiterate those of others ^2,11,12^ regarding the statistically significant and clinically meaningful benefits of engaging people with type 2 diabetes in DSME.

However, the study team found that the addition of telephonic engagement concurrent with DSME did not affect outcomes for people with diabetes significantly. There are several factors that may have contributed to the statistically significant improvements in both groups. The sites selected to participate in this study had to have had a sufficiently robust, active, and high–quality DSME program to guarantee enrollment of study participants. All 4 FQHCs had level 2 or 3 recognition from the National Committee for Quality Assurance (NCQA) and, in addition, had received accreditation status by either AADE or the American Diabetes Association. Accreditation by these organizations ensures compliance with the National Standards for Diabetes Self-Management Education and Support.^3^ In addition, these programs already included a peer support component, which has been suggested to be helpful for improving clinical outcomes of people with type 2 diabetes.^13^

FQHCs serve economically disadvantaged populations. They also commonly use the patient-centered model of care delivery, have an appearance of patient loyalty, and at least with those attaining level 3 NCQA recognition, show greater improvement across a number of outcomes.^14^ This might suggest that patients cared for in such settings might not benefit from the addition of support. The time frame of 18 months may have been too short to result in a change in outcomes.^13^

It is important to note that those receiving the telephonic support did have a significantly greater number of visits (Table 3). The study team interprets this to mean that telephonic support reinforced the importance of obtaining risk-reducing care.

Qualitative insight gleaned from study participant discussions with their diabetes educators was that those receiving the telephonic support were appreciative of the support. This may suggest that telephonic support of this type may be especially important in cases when individuals cycle between contemplating and preparing to incorporate lifestyle changes.^14,15^ The model of providing telephonic support using a centralized call center as an approach to helping individuals get to a point where they are ready to engage in self-care is a model that should be explored further.

Many in the intervention group commented that they were sad that the telephonic support had to end. The telephonic support provided in this study was concurrent with at least a portion of the DSME participation of study participants. As a chronic disease, managing type 2 diabetes is an ongoing process. Telephonic support is an opportunity to engage with the person with diabetes as a way to enable them to succeed with their goals and identify and solve problems as they encounter challenges. All 4 FQHCs are continuing to support their patients with diabetes through telephone, email, or texting after the study, indicating that they saw value in this intervention.

The AADE accreditation principles utilized in this randomized, controlled evaluation contributed to the delivery of comprehensive face-to-face care with consistent assessment and documentation over time. The 4 FQHCs in this study executed these principles effectively with resultant improvement in glycemic status in a large group of patients with an average HbA1c >10 at enrollment. Results show that this patient-centered, team-based care effectively produced statistically significant and clinically meaningful improvement for all participating patients, with 12-month HbA1c reductions of 1.7 and 1.4 for intervention and control groups, respectively. Diabetes is a chronic condition and as such requires ongoing engagement. There remains a need to identify additional avenues to provide support, perhaps through innovative involvement with diabetes educators to address unmet needs,^15^ improved engagement with peers through social media,^16,17^ or novel touchpoints to reinforce messaging through either previously untapped health care or community settings.

This randomized, controlled study demonstrated that comprehensive face-to-face care with consistent assessment and documentation over time produced significant improvement for people with diabetes. The addition of structured telephonic support overlapping in time with the comprehensive face-to-face provision of care in this environment did not produce statistically significant clinical or behavioral care improvement. It is worth noting that the professionals who work with the people with diabetes found value in the added connection. Perhaps telephonic support in settings that do not offer comprehensive DSME would help maintain continuity of care with their people with diabetes. This could apply both within FQHCs and other health care settings. Further research and exploration of means to enhance DSMES are needed to advance the understanding of approaches that will advance care.
